# Abnormal joint torque patterns exhibited by chronic stroke subjects while walking with a prescribed physiological gait pattern

**DOI:** 10.1186/1743-0003-5-19

**Published:** 2008-09-01

**Authors:** Nathan D Neckel, Natalie Blonien, Diane Nichols, Joseph Hidler

**Affiliations:** 1 Center for Applied Biomechanics and Rehabilitation Research (CABRR), National Rehabilitation Hospital, 102 Irving Street, NW, Washington, DC 20010, USA; 2 Physical Therapy Service, National Rehabilitation Hospital, 102 Irving Street, NW, Washington, DC 20010, USA; 3 Department of Biomedical Engineering, Catholic University, 620 Michigan Ave., NE, Washington, DC 20064, USA

## Abstract

**Background:**

It is well documented that individuals with chronic stroke often exhibit considerable gait impairments that significantly impact their quality of life. While stroke subjects often walk asymmetrically, we sought to investigate whether prescribing near normal physiological gait patterns with the use of the Lokomat robotic gait-orthosis could help ameliorate asymmetries in gait, specifically, promote similar ankle, knee, and hip joint torques in both lower extremities. We hypothesized that hemiparetic stroke subjects would demonstrate significant differences in total joint torques in both the frontal and sagittal planes compared to non-disabled subjects despite walking under normal gait kinematic trajectories.

**Methods:**

A motion analysis system was used to track the kinematic patterns of the pelvis and legs of 10 chronic hemiparetic stroke subjects and 5 age matched controls as they walked in the Lokomat. The subject's legs were attached to the Lokomat using instrumented shank and thigh cuffs while instrumented footlifters were applied to the impaired foot of stroke subjects to aid with foot clearance during swing. With minimal body-weight support, subjects walked at 2.5 km/hr on an instrumented treadmill capable of measuring ground reaction forces. Through a custom inverse dynamics model, the ankle, knee, and hip joint torques were calculated in both the frontal and sagittal planes. A single factor ANOVA was used to investigate differences in joint torques between control, unimpaired, and impaired legs at various points in the gait cycle.

**Results:**

While the kinematic patterns of the stroke subjects were quite similar to those of the control subjects, the kinetic patterns were very different. During stance phase, the unimpaired limb of stroke subjects produced greater hip extension and knee flexion torques than the control group. At pre-swing, stroke subjects inappropriately extended their impaired knee, while during swing they tended to abduct their impaired leg, both being typical abnormal torque synergy patterns common to stroke gait.

**Conclusion:**

Despite the Lokomat guiding stroke subjects through physiologically symmetric kinematic gait patterns, abnormal asymmetric joint torque patterns are still generated. These differences from the control group are characteristic of the hip hike and circumduction strategy employed by stroke subjects.

## Background

Following stroke, individuals may experience weakness [1-4], changes in passive joint properties [5], spasticity [6-8] and or altered muscle coordination [4,9-11]. In the lower limbs, these impairments lead to walking deficits such as decreased endurance [12], slower gait speed [13] or an asymmetrical gait cycle [14]. Since asymmetric patterns are often equated to poor stability during gait which increases the risk for falls [15], restoring gait symmetry is often the goal of rehabilitative gait training. For example, during body weight supported treadmill training, hemiparetic stroke subjects often produce a more symmetrical gait pattern [16]. And it has been shown that symmetrical gait patterns can be temporally induced in stroke subjects after walking on a split belt treadmill with each belt running at a different speed [17].

An additional approach that may enable stroke subjects to walk symmetrically is with the use of robotics. The Lokomat robotic orthosis is a device that guides a subject through a symmetric physiological gait pattern as they walk on a treadmill with or without body weight support [18,19]. While gait training with the Lokomat has shown the ability to improve the walking performance of acute stroke subjects in clinical scales [20] and step length [21], it is unclear whether symmetric kinematic training also results in symmetric joint torques and muscle activation patterns which underlie locomotion.

Unfortunately the joint torque patterns of stroke subjects are poorly understood, most likely due to the practical difficulties associated with repeatedly testing stroke subjects in the modern gait laboratory. Previous studies have shown that stroke subjects exhibit greater knee flexion during pre-swing [22] as well as greater peak ankle dorsiflexion torque and hip flexion torque during stance [23]. But these studies are based on no more than 5 non-consecutive steps, sometimes with the aid of a cane, or only collecting data from one limb at time. To more accurately quantify representative post-stroke kinetics, a large number of steps equally collected from of a wider range of impairment levels is required.

The goal of this study was to determine whether chronic, hemiparetic stroke subjects that are guided through symmetric kinematic trajectories are capable of generating symmetric joint torques and muscle activation patterns. For this study, advanced instrumentation has been added to the Lokomat that allows for the estimation of joint torques throughout the gait cycle while subjects walk in the device [24]. A split belt instrumented treadmill was used to capture the ground reaction force of each separate leg, multi-degree of freedom load cells attached to the Lokomat leg cuffs and force sensors mounted to the foot lifters measured the interaction forces between the subject and the Lokomat, and a motion capture system tracked the location of each limb segment. Using this instrumentation, along with a custom inverse-dynamics algorithm [24], the joint torques and muscle activation patterns stroke subjects exhibit while moving through symmetric kinematic patterns could be identified. Clinically, this information is important for properly interpreting clinical studies involving the Lokomat, and for increasing our understanding of the capacity of hemiparetic stroke subjects to break out of stereotypical abnormal lower limb motor behaviors that are often employed to compensate for lower limb impairments.

## Methods

### Subjects

Ten chronic hemiplegic stroke subjects (age: 51–65, avg 56.5 yrs, SD 4.9) with mild to moderate lower limb impairments (Fugl-Meyer lower limb scores 16–31 avg 21.1, SD 5.3) were tested along with five healthy subjects with no known neurological impairments or gait disorders (age: 51–69, avg 58.8, SD 6.7). Stroke inclusion criteria included unilateral lesion of the cortex or subcortical white matter with an onset greater than one year prior to testing. Subjects were excluded from the study if they presented with severe osteoporosis, contracture limiting range of motion, significant muscle tone, cardiac arrhythmia, or significant cognitive or communication impairment which could impede the understanding of the purpose of procedures of the study (less than 24 on the Mini Mental State Exam [25]). All experimental procedures were approved by the Institutional Review Boards of Medstar Research Institute and the Catholic University of America. Informed consent was obtained prior to each test session.

Motor impairment was evaluated in the paretic lower extremity using the Fugl-Meyer (FM) scale [26], which ranges from 0 to 34 with the maximum score indicating no observable deficits in function. In order to study hemiparetic stroke patients with mild to moderate impairment levels, we targeted subjects having a FM score in the range of 10–30.

### Instrumentation

A Codamotion active marker system (Charnwood Dynamics LTD, UK) was used to track the leg kinematics of each subject in the same manner as Neckel and Hidler [27]. Tracking kinematic patterns using a motion capture system was necessary since subject's legs are not rigidly coupled to the Lokomat and therefore do not move through the same trajectory as the system's linkages [28]. Thus relying on the Lokomat potentiometers to measure leg kinematics is highly inaccurate. Custom marker clusters were used such that the cuffs that fix the subject to the Lokomat would not interfere with the placement of the 24 active markers used. First, rigid plastic bases with foam undersides were inserted under the Lokomat leg cuffs. The motion tracking marker clusters were then fixed to rigid plastic caps that fit firmly on top of both the base and Lokomat leg cuff strap with Velcro straps. The Codamotion camera was placed approximately 2 meters in front of the Lokomat. The marker positions were recorded at 100 Hz and exported to the software package Visual 3D (C-Motion INC, Rockville MD) where a customized model of each subject was created from anthropometric data. From this model limb segment center of mass, segment acceleration, joint centers and limb angles were derived and exported to the software package Matlab (Mathworks, Natick MA) for further filtering and processing.

**Figure 1:**
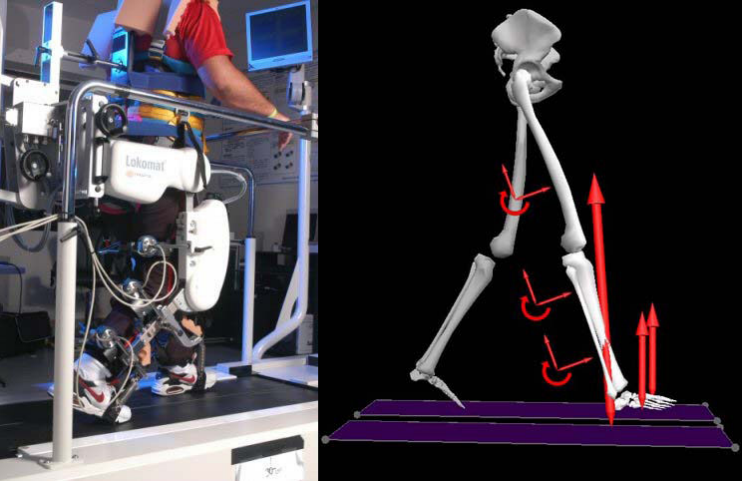
**Setup of instrumentation**. The photograph on the left shows the loadcells on the leg cuffs of the Lokomat which measure the interactions between the subject and the device. The graphic on the right represents the recorded forces acting on a subject's right limb – ground reaction force, footstraps, and loadcells. Graphic adapted from Visual 3D (C-Motion INC, Rockville MD).

An ADAL split-belt instrumented treadmill (TECHMACHINE, Andrézieux France; see Belli et al., 2001 for detailed description [29]) was used below the Lokomat, which allowed for ground reaction forces to be recorded for each leg in the vertical, anterior-posterior, and medial-lateral axes. Each of the six Lokomat cuff brackets that couple the subject's leg to the device were instrumented with 6 degrees of freedom loadcells (JR3 Inc, Woodland CA) that measured the interaction forces and torques applied to the subject's legs by the Lokomat. The Lokomat is equipped with optional footstraps that lift the forefoot up so that the toes can clear the ground during swing. These footstraps were used on the affected leg of all stroke subjects, where the tension in each strap was measured with uniaxial force sensors (MLP-50, Transducer Techniques, Temecula CA). A photograph of the loadcell setup along with a schematic of the measured forces can be seen in Figure 1.

Electromyographic (EMG) recordings were collected from the tibilias anterior, gastrocnemius, biceps femoris, vastus medialis, rectus femoris, gluteus maximus, gluteus medius, and adductor longus of both limbs in stroke subjects and the left limb of four of the five control subjects (one subject was improperly grounded and their EMG data was not analyzed) using two Bagnoli-8 EMG system (Delsys, Inc., Boston, MA). EMG data, along with the forces and torques from the loadcells, were anti-alias filtered at 500 Hz prior to sampling at 1000 Hz using a 16-bit data acquisition board (Measurement Computing, PCI-DAS 6402, Middleboro, MA) and custom data acquisition software written in Matlab and stored for later analysis. Force plate data was further low-pass filtered using a zero-delay fourth order Butterworth filter with a 25-Hz cutoff frequency.

### Protocol

The stroke subjects were first fitted with a harness so that a portion of their body-weight could be supported while control subjects did not wear the harness. Subjects were led into the Lokomat and with the help of a physical therapist the device was adjusted so that the Lokomat hip and knee centers lined up with those of the subject. After being correctly aligned, the marker clusters were applied to the subject's feet, shanks, and thighs. A neoprene band was tightly wrapped around the subject's waist and individual motion tracking markers were affixed to the boney landmarks of the pelvis.

After the subject was in the Lokomat, an experienced physical therapist conducted a practice session for up to 2–3 minutes to allow the subject to acclimate to the device. Stroke subjects began walking suspended above the treadmill and the amount of body weight support provided by the accurate and constant Lokolift system [30] was reduced until a minimum level that produced an appropriate gait pattern was found. Inappropriate gait patterns were judged by the physical therapists and included such factors as impaired limb buckling during stance, toe dragging through swing, and excessive trunk movements that would not be analogous to a healthy gait pattern. The levels of minimum body weight support ranged from 11.5 to 25.6 percent of total body mass.

Following the acclimation period, the speed of the Lokomat was randomly adjusted to one of 4 different speeds (1.5, 2.0, 2.5, and 3.0 km/hr), and after allowing the subject to acclimate to the new speed 30-seconds of data was collected. The subject was told to try and match the kinematic pattern of the Lokomat to the best of their ability. It should be noted that the Lokomat was run with 100% guidance force under these trials, meaning the device was in a pure position control mode rather than an impedance mode. While the Lokomat has the ability to change the amount of subject assistance, our goal was to determine whether subjects assisted through physiological gait patterns produce symmetric, normal joint torques. For this, position control mode was more appropriate than an impedance mode. The remaining 3 speeds were tested in the same manner. Adequate rest breaks were taken throughout the experiment to minimize fatigue. For the purposes of this paper, only trials run at 2.5 km/hr are reported.

Following all trials, a precision digitizing arm (MicroScribe MLX, Immersion, San Jose CA) was used to accurately locate the position of the Lokomat, load cells, and foot lifter locations with respect to anatomical landmarks. This information was necessary to determine the location of the Lokomat forces acting on the subject's lower extremities when computing the joint torques throughout the gait cycle [24].

### Data analysis

The vertical ground reaction forces were used to mark the heel strike of each step, measured as the point were the force exceeded 50 N. All experimental data (including that calculated in Visual 3D) over the 30-second trials were broken up into individual strides (from heel strike to heel strike in the same leg), which were then resampled to the same signal length. The subject kinematics calculated from Visual 3D (limb segment center of mass location, segment acceleration, joint center locations and limb segment locations) were combined with all the forces and torques acting on the subject – the ground reaction forces from the split-belt instrumented treadmill, as well as at the Lokomat leg cuffs (location of the loadcells calculated from the Lokomat potentiometers and digitized Lokomat limb lengths) – into a custom inverse dynamics model [24]. This model was then used to calculate joint torques that the subjects were generating throughout the trial in both the frontal and sagittal planes, as well as the torques that the Lokomat were inducing on the subject. For each subject, the data generated for all steps within a 30-second trial was averaged for each limb.

### Statistical analysis

A total of 5 kinematic and 5 kinetic measures of the profiles of the impaired, unimpaired, and control limbs (left limb) were compared using a single factor ANOVA. The kinematic measures were ankle, knee and hip range of motion (ROM), maximum vertical pelvic displacement from heelstrike, and the time in the gait cycle at which the minimum pelvic displacement occurred. The kinetic measures were maximum vertical ground reaction force, maximum ankle dorsiflexion torque, magnitude of knee extension torque at the midpoint of the initial swing phase (68.5% gait cycle), the time at which the maximum hip extension torque occurred, and the magnitude of the hip adduction torque at mid swing (80% gait cycle). A Bonferroni correction was used to reduce the risk of Type I errors, so that with 10 measures tested, a α = 0.005 was used for all comparisons.

The EMG activity from the selected muscle groups was band-pass filtered (20–450 Hz), full-wave rectified, and then smoothed using a 200-point RMS algorithm. For each muscle recorded, the EMG traces were normalized to that subject's highest value recorded across all trials to allow for inter-subject comparison. The mean normalized EMG trace for each subject was broken up into seven phases of the gait cycle (initial loading 0–12%, mid-stance 12–30%, terminal-stance 30–50%, pre-swing 50–62%, initial-swing 62–75%, mid-swing 75–87%, terminal-swing 87–100%) and each section integrated as in Hidler and Wall [31].

## Results

### Kinematics

**Figure 2:**
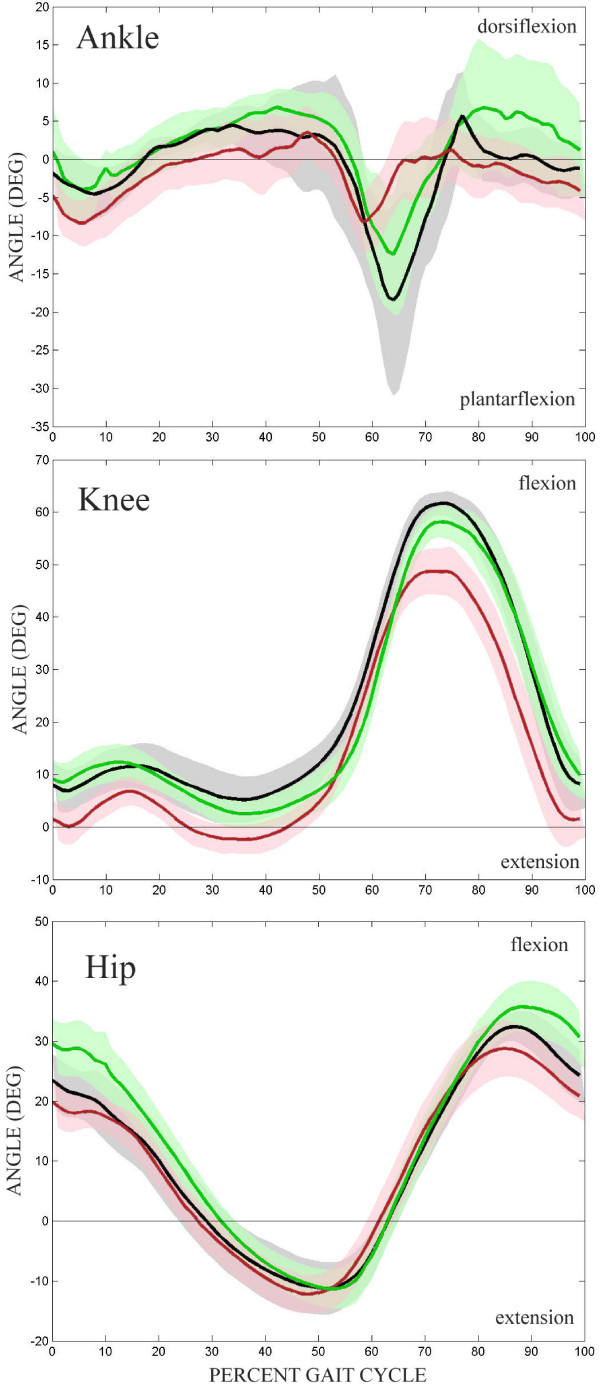
**Joint kinematics**. Mean sagittal joint angles of the ankle, knee, and hip through the gait cycle (from heelstrike to heelstrike). Control – black, unimpaired – green, impaired – red. Shaded region represents 95% CI.

The mean ankle, knee and hip sagittal plane joint angles for all three limbs tested (impaired, unimpaired, control) are shown in Figure 2 with specific values found in Table 1. In general, there were only slight differences in the kinematic patterns exhibited between the control subjects and the impaired and unimpaired limbs of the stroke subjects. At toe-off control subjects had a larger peak plantarflexion angle than either stroke ankle, but the impaired ankle was slightly more plantarflexed throughout the rest of the gait cycle. The knee angles were quite similar, although the impaired knee tended to be slightly more extended through the gait cycle, resulting in a peak flexion angle through swing that was lower than either the unimpaired or control limb. The hip angles were similar as well, with the impaired hip being more extended throughout the gait cycle, and the unimpaired hip being more flexed, especially terminal swing and initial loading.

**Table 1:** Mean kinematic measures.

	Control	Unimpaired	p vs Control	Impaired	p vs Control	p vs Unimpaired
Ankle ROM	28.53 (4.96)	28.98 (1.80)	0.918	17.75 (1.80)	0.025	<.001*
Knee ROM	57.21 (1.64)	57.07 (1.34)	0.952	53.18 (2.32)	0.273	0.164
Hip ROM	44.47 (1.60)	48.38 (2.25)	0.273	41.84 (1.89)	0.387	0.039
Time of Pelvis Min	3.79 (1.36)	4.16 (0.45)	0.750	7.97 (1.22)	0.056	0.009
Pelvis Max	0.89 (0.11)	1.23 (0.20)	0.260	0.76 (0.11)	0.476	0.050

Standard error of the mean in parenthesis. * represents significant difference (p < .005).

**Figure 3:**
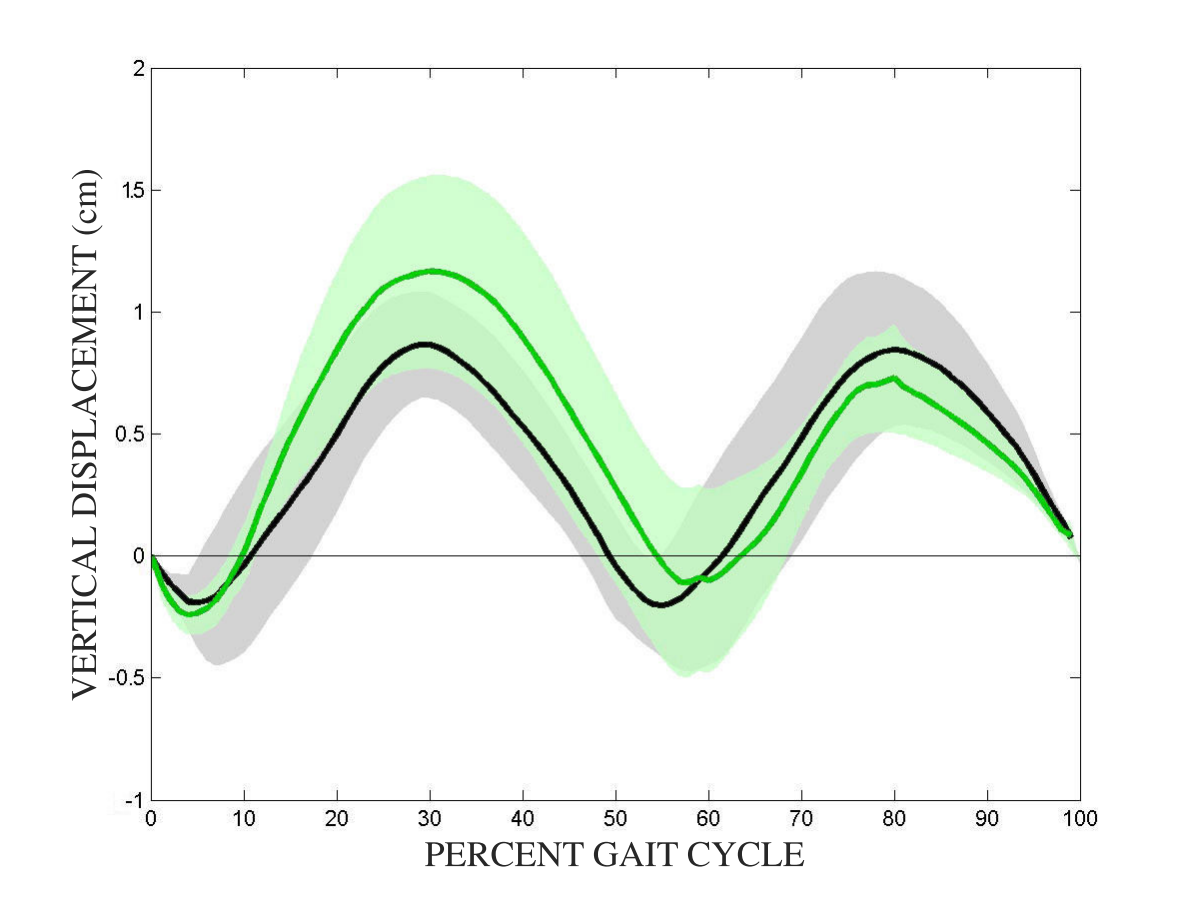
**Pelvic motion**. Mean vertical displacement of the pelvis center of gravity from heelstrike and single support of the left/unimpaired limb to double limb support, and finishing with single limb support on the right/impaired limb. Shaded region represents 95% CI.

Figure 3 shows the mean vertical displacement of the pelvis center of gravity of the stroke and control groups from heelstrike of the left leg (control) or unimpaired leg (stroke) to single support on the left/unimpaired limb, then to double limb support, and finishing with single limbs support on the right/impaired limb. The pelvis of stroke subjects consistently raised up higher during unimpaired limb support than during impaired limb support, and the minimum pelvic height following unimpaired limb support comes later in the gait cycle than the minimum pelvic height following normal single limb support. The frontal plane angles were also derived and in general, there was very little movement in the frontal plane, and no differences between the three limbs tested.

Table 1 lists the average value, standard error of the mean, and p-values for the 5 kinematic measures tested. There were no significant kinematic differences between the control limb and the unimpaired limb of stroke subjects, no significant differences between the impaired limb and control limb, and only 1 significant difference between the impaired and unimpaired limb (ankle ROM).

### Kinetics

**Figure 4:**
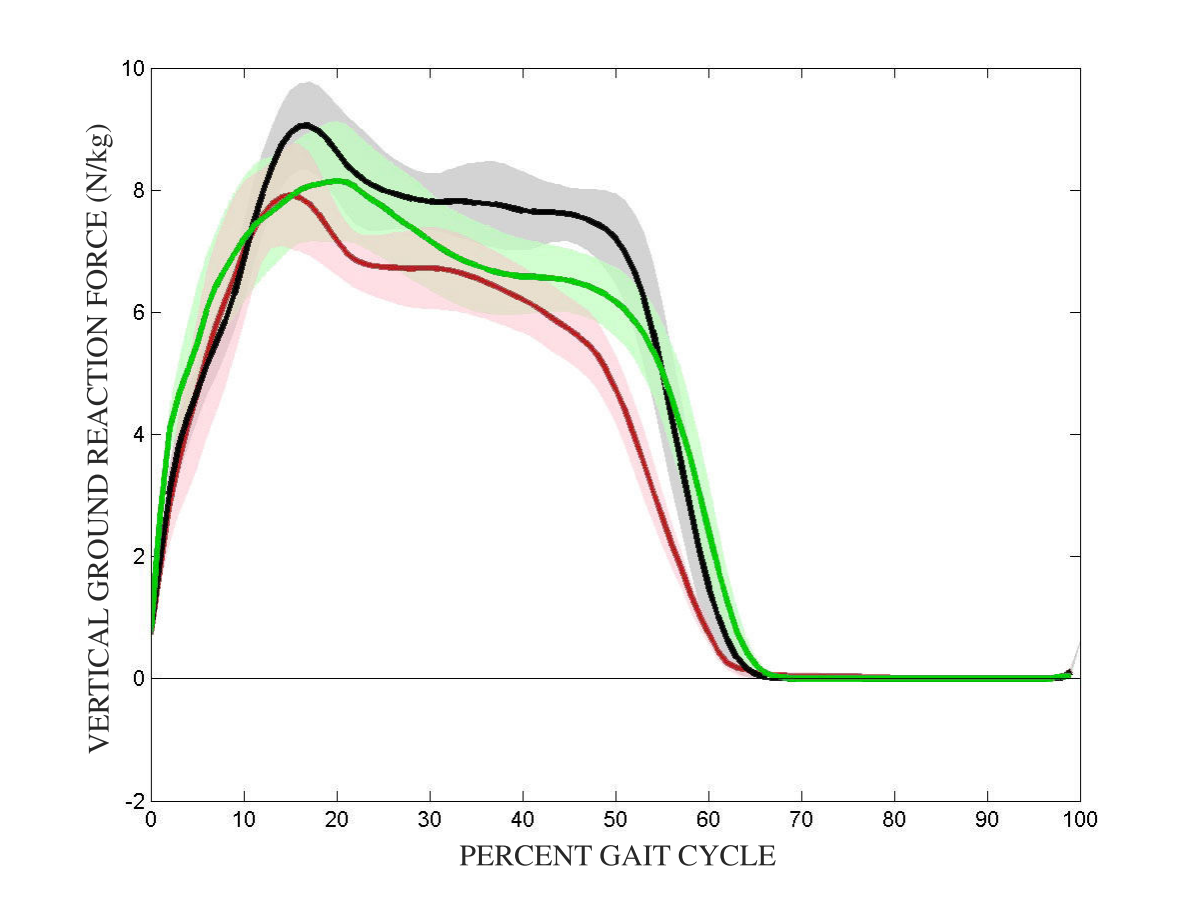
**Ground reaction force**. Mean vertical ground reaction force through the gait cycle. Control – black, unimpaired – green, impaired – red. Shaded region represents 95% CI.

The mean vertical ground reaction forces (GRFs) throughout the gait cycle of the impaired, unimpaired, and control limbs are presented in Figure 4. For both the control and stroke subjects, the vertical GRFs did not demonstrate the classic double bump throughout stance. Since the Lokomat is supported on a parallelogram that is supported by a large spring, the Lokomat maintains continuous upward lift to the subject through stance. While all three traces follow similar paths for the 3 limbs, the ground reaction force of the impaired limb tended to be lower in magnitude than the unimpaired limb, which in turn was less than the control. None of these differences reached the significant level, presumably due to the large variability in these measures.

**Figure 5:**
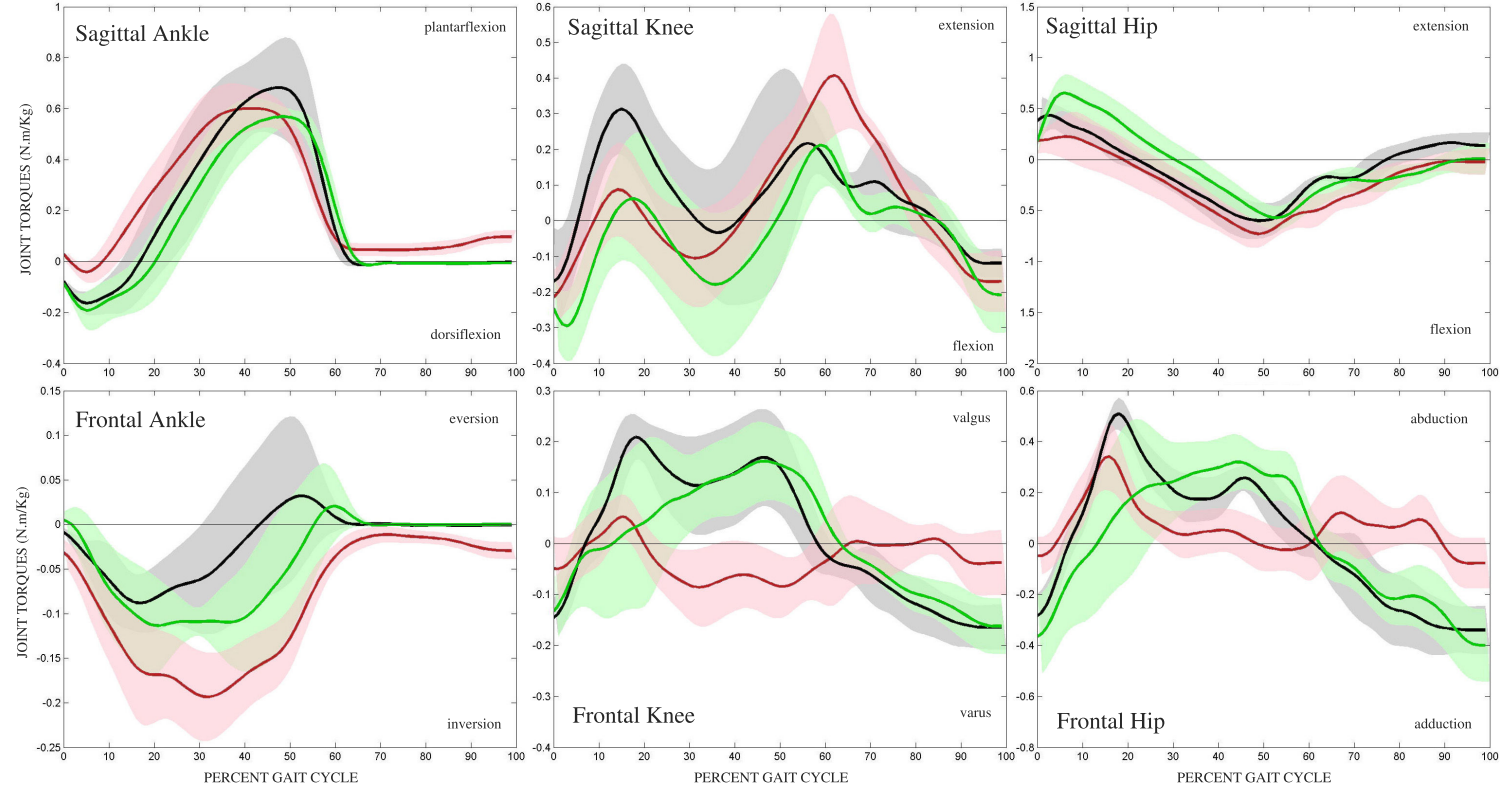
**Joint kinetics**. Mean sagittal (top) and frontal (bottom) joint torques of the ankle, knee, and hip through the gait cycle. Control – black, unimpaired – green, impaired – red. Shaded region represents 95% CI.

The mean sagittal and frontal plane joint torques for the ankle, knee, and hip for all three limbs as they progress through the gait cycle is shown in Figure 5. Upon general visual inspection, the sagittal ankle torques of the unimpaired and control limb follow very similar patterns, whereas the sagittal ankle torque in the impaired limb of stroke subject was quite different, with less dorsiflexion at initial contact and continuous ankle extension during swing. The diminished dorsiflexion results from the subject wearing the foot lifter, which reduces the need to flex the ankle as it makes contact with the treadmill belt. Similarly, the continuous active ankle extension torque during swing results from the subject trying to extend their ankle to a more neutral position. In the frontal plane, stroke subjects exhibited larger eversion torques during stance in both limbs. Neither of these torque profiles were similar to the frontal plane torques in the control subjects, where controls had a lower eversion torque during early to mid stance and an inversion torque during late stance and toe-off.

The knee torques generated in the sagittal plane in both the impaired and unimpaired knees of the stroke subjects follow similar patterns during stance, with lower extension torques than the controls in early stance. In mid-stance, stroke subjects tend to flex their knees to a greater extent than controls in both limbs. From toe-off through swing, the unimpaired limb behaved similar to the control limbs, but the impaired limb demonstrated a consistent, large extension torque at toe-off that is higher than both the control and unimpaired limbs. In the frontal plane, the unimpaired knee behaves similar to the control knee but, with slightly less varus torque in early-stance. The impaired limb is drastically different than the other two torque profiles, where there were significant valgus torques during mid to late stance as well as less valgus through swing.

All 3 sagittal hip torques follow very similar patterns with a few noteworthy differences. The maximum extension torque was much greater in the unimpaired limb than the impaired limb in early stance, peaking later in the gait cycle that either of the other limbs. Surprisingly, the average maximum flexion torque was greater in the impaired limb than the unimpaired limb or the control subjects at the end of stance. In the frontal plane the impaired stroke limb produced less abduction during stance and more abduction during early to mid-swing than either the unimpaired or control limbs.

Table 2 lists the average value, standard error of the mean, and p-values for the 5 kinetic measures tested. There were no significant differences between the control limb and the unimpaired limb of stroke subjects, 3 significant differences between the impaired limb and control limb (maximum ankle dorsiflexion, knee extension at initial swing, and hip adduction at mid swing), and 3 significant differences between the impaired and unimpaired limb (maximum ankle dorsiflexion, knee extension at initial swing, and hip adduction at mid swing).

**Table 2:** Mean kinetic measures.

	Control	Unimpaired	p vs Control	Impaired	p vs Control	p vs Unimpaired
GRF max	9.09 (0.36)	8.75 (0.49)	0.658	8.65 (0.41)	0.506	0.884
Ankle Flexion	0.18 (0.03)	0.23 (0.04)	0.446	0.06 (0.02)	0.003*	0.001*
Knee Extension Initial Swing	0.10 (0.03)	0.03 (0.03)	0.097	0.27 (0.02)	0.001*	<.001*
Time of max Hip Extension	3.02 (1.15)	6.61 (0.87)	0.030	3.12 (1.31)	0.963	0.04
Hip Adduction Mid Swing	0.26 (0.07)	0.22 (0.06)	0.613	-0.07 (0.04)	0.001*	0.001*

Standard error of the mean in parenthesis. * represents significant difference (p < .005).

### EMG

**Figure 6:**
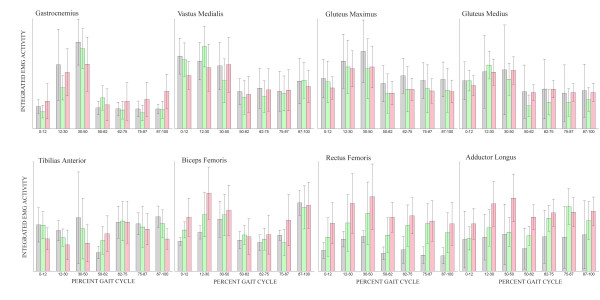
**Muscle activity**. Integrated mean normalized EMG values over the seven gait phases. Control – black, unimpaired – green, impaired – red. Error bar represents standard error of the mean.

The mean integrated muscle activity of the eight muscle groups for the impaired, unimpaired, and control limbs over the seven phases of the gait cycle are shown in Figure 6. For the most part, the three groups behave quite similarly. In the gastrocnemius the impaired limb had slightly higher activity during swing. In the tibilias anterior, mean activity was 41% less in the impaired limb compared to controls at terminal swing. During initial contact, the biceps femoris on the impaired limb was slightly higher and the vastus medialis was slightly lower than the other two groups, but through the rest of gait these muscles were very similar to unimpaired and control levels. There were no notable differences between the gluteus medius activity of the three groups. The rectus femoris in the impaired limb had a level of activity that was consistently higher than the other two limbs (e.g. unimpaired and controls), this mean activity was at least 2.6 times higher than the control group from pre-swing through terminal swing. This higher level of activity in the impaired limb was also true in the adductor longus, but here the impaired values were at least 53% higher than the unimpaired values during terminal stance and pre-swing, and 2.4 times greater than controls during pre-swing. All three groups produced similar levels of gluteus maximus activity throughout the gait cycle.

### Lokomat torques

**Figure 7:**
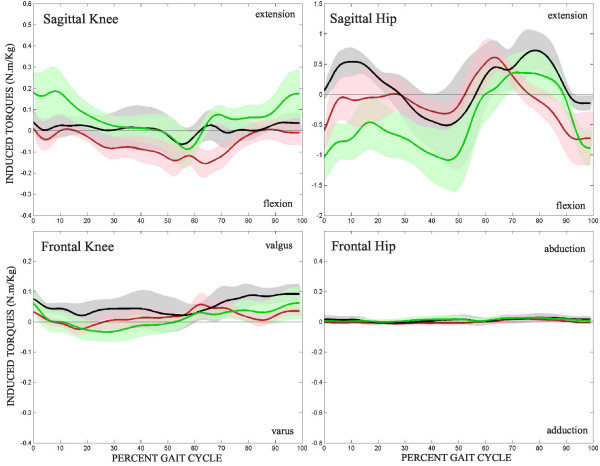
**Robot induced torques**. Mean torques induced at the joints by the Lokomat through the gait cycle. Control – black, unimpaired – green, impaired – red. Shaded region represents 95% CI. Vertical axis are scaled to match corresponding subject generated torques of figure 5.

The mean sagittal and frontal torques induced by the Lokomat at the subject's knee and hip throughout the gait cycle are shown in Figure 7. The Lokomat consistently induces low levels of torque in the frontal plane of all three subject groups at the hip and knee, but there is notable variability in the sagittal plane.

At the knee, the control subjects experience low levels of sagittal knee torque from the Lokomat throughout the gait cycle. However, the unimpaired limb experiences a greater extension torque at the knee from the Lokomat during terminal swing and initial contact than either the impaired or control limbs. The impaired limb experiences a greater flexion torque at the knee from the Lokomat during late stance and pre-swing than the unimpaired and control limbs.

At the hip, the Lokomat imparts an extension torque on the control hip that starts at heelstrike, peaks during early stance, and returns to zero by mid-stance. From mid to late stance, the Lokomat produces a flexion torque on the control subjects, followed by another slightly larger extension torque peak through swing. Both stroke limbs feel this peak-valley-peak of Lokomat-induced torque at the hip but it is shifted during early stance. Instead of an extension torque being imparted on the hip by the Lokomat, the stroke limbs feel decreasing flexion torques. Beyond mid stance, the impaired limb feels similar torques from the Lokomat as the control subjects, but the unimpaired hip feels a much higher level of flexion at pre-swing and terminal swing. Statistical differences between the induced Lokomat torques on the three limbs were not found.

## Discussion

The joint angles that both limbs of stroke subjects produce while walking in the Lokomat are similar to healthy subjects, which is not surprising considering the Lokomat guides subjects through a prescribed kinematic pattern. Even at the ankle, which is not driven by the Lokomat, subjects produce similar overall patterns in the sagittal plane. Despite the kinematic patterns being similar, stroke subjects still generated abnormal joint torque patterns, particularly in the impaired limb. Many of these abnormal patterns are consistent with the clinical characteristics of overground hemiparetic gait. Evidence of hip hiking, stiff legged gait with impaired limb circumduction, and asymmetric limb support times are still present in the Lokomat, suggesting the inability of hemiparetic stroke subjects to break out of stereotypical abnormal motor behaviors even when the movement tasks are simplified.

The fact that there are no significant kinematic differences between the stroke limbs and the control limbs can be attributed to the fact that the Lokomat is guiding the limbs of all subjects through pre-programmed trajectories. We have previously shown that despite being firmly strapped into the Lokomat, subjects retain the ability to move and shift relative to the device [28]. Nevertheless, the angles produced at the ankle, hip, and knee are quite similar, even for the impaired limb of stroke subjects. The lone kinematic difference is the ankle ROM between the impaired limb and unimpaired limb. Since the Lokomat does not drive the ankle, passive footstraps are often applied to the impaired ankle, which assist dorsiflexion during swing yet limit the extent of ankle plantarflexion. It was expected that these footstraps would limit ROM for the impaired limb compared to both the control and unimpaired limbs. Surprisingly, differences were only significant when compared to the unimpaired limb and not the control ankle ROM.

While there were no significant differences found for the knee or hip kinematic patterns, the data presented in Figure 2 show some noteworthy characteristics. The differences between the knee impaired limb and both the control or unimpaired limb can be explained in part by stroke subjects trying to hyperextend the impaired knee while standing. If stroke subjects comfortably stand with the impaired limb in hyperextension, and are then set-up in the Lokomat as such, the sagittal kinematic pattern of the knee will be shifted slightly in extension. The average difference between the unimpaired and impaired limbs was 5 degrees, noticeable, but possibly clinically insignificant. The kinematic differences at the hip between the unimpaired and impaired limb can be attributed to stroke subjects attempting to get off of the impaired limb and quickly back onto the unimpaired limb during normal ambulation. In the Lokomat, this strategy manifests as a lower range of motion of the impaired hip, and the flexion and extension peaks appear to be reached at an earlier part of the gait cycle.

The pelvic vertical displacement of healthy subjects traveled in the standard symmetric sinusoidal pattern, while the pelvis of stroke subjects was higher during the unimpaired single limb support phase than during the impaired single limb support phase. This is consistent with the common strategy of stroke subjects who hike the hip up while on the unimpaired limb in order to have enough clearance for the impaired limb to swing through. One may believe that since the Lokomat imparts a physiological kinematic pattern that this hip hiking would be unnecessary. However as described in the results section, even though the stoke subject's kinematics are being guided by the Lokomat, they still have a tendency to extend their knee in pre-swing and also abduct their hip in mid swing. Both of these stereotypical strategies result in hip hiking as we saw in Figure 3. The Lokomat further exacerbates this behavior since subjects cannot shift their weight to the contralateral limb nor can they abduct their leg to help with toe clearance. As a result, subjects tend to hip hike for toe clearance. It is also worth mentioning that the valleys of the pelvic traces that represent double limb support are farther apart in the gait cycle between unimpaired limb single stance than impaired limb single stance (not significantly different, p=.009), again, consistent with the habit of spending more time on the unimpaired limb.

As can be seen from the Table 2 there are no significant kinetic differences between the unimpaired limb and the control limb for the measures tested here. The vertical ground reaction forces of all three limbs lack the characteristic double peak, which is typical in the Lokomat [24]. Even with a minimal amount of body weight support, the vertical ground reaction forces of the stroke limbs were lower, but not significantly different. Generally, stroke subjects do not (or cannot) generate enough hip extension torque to carry the pelvis over the impaired limb while it is in stance, and thus rush the gait cycle while on the impaired limb and extend the gait cycle while on the unimpaired limb. This behavior is still present during Lokomat-assisted treadmill walking. The unimpaired hip reaches its maximum extension later during early stance than the controls or impaired limb; this may be due to stroke subjects taking more time to vault the pelvis over the unimpaired limb as the impaired limb goes through swing. From Figure 3 it can be seen that the hip extension torque during early stance of the impaired limb is lower than that of the unimpaired limb. This can again be explained by the stroke subject's tendencies to not fully extend the impaired hip to pull the pelvis forward but to quickly get off of the impaired limb during overground ambulation.

From Figure 3 it can be seen that the control subjects reach maximum hip abduction during mid-stance while the unimpaired limbs reach maximum hip abduction during late stance. If hip abduction torques are present to keep the pelvis level during single limb support, this may help explain the differences in hip abduction torques. If the impaired limb is slow to enter swing, it could be rationalized that the maximum abduction torque of the unimpaired limb would occur later. It was also observed that the unimpaired limb exhibits greater knee flexion during early stance. This again may be to help vault the pelvis over the unimpaired limb.

In the frontal plane, the stroke subjects lack any appropriate adduction torques of the impaired limb. Both the control and unimpaired limbs adduct the hip during swing and early stance, presumably to keep the limb within the workspace of the Lokomat and treadmill. But the impaired limb does the opposite and abducts or pushes against the Lokomat through swing. This behavior is likely a strategy to overcome a lack of knee flexion during swing by circumducting the impaired limb outward to achieve toe clearance through swing. While the Lokomat resists much of this motion, subjects are able to over-power the device slightly and achieve some out of plane movement. This abnormal knee behavior of the impaired limb presents itself as a significantly excessive extension torque during initial swing. Again, this is part of the circumduction strategy of stroke subjects, where the knee is extended or stiffened before the whole limb is swung out and around during swing.

The impaired ankle exhibits little dorsiflexion and very quickly transfers to plantarflexion during stance. This lack of dorsiflexion may be due to the fact that the footlifter is flexing the ankle for the stroke subject, and there is no need for voluntary dorsiflexion of the impaired ankle. Throughout stance of healthy over ground gait, the ankle everts as a mechanism to prevent outward rolling of the ankle. But in the Lokomat, we have found that healthy subjects will invert during late stance and toe-off, presumably in response to the lack of lateral sway of the hips above and the need to propel the limb straight forward through swing. Both ankles of stroke subjects do not behave in this manner in the Lokomat, instead producing eversion torques during stance similar to healthy over ground walking. The unimpaired limb will produce a small inversion torque at toe-off, but from Figure 3 this force appears to be smaller and occur later in the gait cycle than in the control population.

The source of some of the abnormal torque behaviors of stroke subjects during Lokomat assisted treadmill walking can be traced back to the muscle activation patterns. The lower tibilias anterior activity during early stance and higher gastrocnemeous activity during swing can account for the different sagittal torque profile of the impaired ankle. Unfortunately the excessive impaired knee extension during toe off does not show up as a higher vastus medialias activity, yet could result from other knee extensor muscles that were not recorded from. A trend that may explain the low knee extension of both the impaired and unimpaired limbs during mid stance is the higher biceps femoris activity, possibly causing antagonistic activity and thus reducing the net torque. The rectus femoris activity of the impaired limbs of stroke subjects is higher throughout the gait cycle than either the unimpaired or impaired limbs but the impaired limb does not exhibit higher hip flexion torques. However, the impaired hip does exhibit lower extension torques, another instance of possible antagonistic activity. Similar findings have been shown in our previous work with stroke subjects performing isometric tasks [3]. And despite high aductor longus activity in the impaired limbs, stroke subjects were not able to generate adequate hip adduction torques. Again, it is possible that other muscles that were not recorded from may have provided some sort of antagonistic behavior. While spasticity can contribute to abnormal torque patterns in the limbs of stroke subjects [32], the subjects tested here were pre-screened by a physical therapist and omitted from the study if they had limited range of motion or significant muscle tone.

With the custom inverse dynamics model used, it was possible to calculate the forces that the Lokomat was applying to the subject's hips and knees throughout the gait cycle. In many cases it can be seen that the Lokomat was trying to augment the torque patterns of the stroke subjects to better match that of the controls. At the knee, the Lokomat induced minimal forces on the control subjects, meaning that the control subjects did a fine job of matching the Lokomat. During late swing and early stance, the unimpaired limb exhibited more knee flexion than the controls and thus the Lokomat produced extension torques about the knee. Similarly, while the impaired knee was producing high extension torque during toe off, the Lokomat tried to correct this behavior by applying a flexion torque. The Lokomat did apply torques to the hips of the control subjects but they are physiologically correct – extension during early to mid stance, flexion from mid stance to toe off and extension through swing. The unimpaired limb of stroke subjects exhibited higher levels of extension and less flexion than the controls through stance and thus the Lokomat was constantly producing a flexion torque on the unimpaired hip through stance. During mid swing the unimpaired limb behaved similar to the control limbs and the Lokomat torques applied to each limb were also similar. Yet at the very end of swing, the Lokomat firmly flexed the hip, but this is not in response to a voluntary extension on the part of the unimpaired limb, for the subjects' hip torques were minimal at this time. It seems as if the stroke subjects simply relaxed and allowed the Lokomat to guide their leg into heel contact. The impaired hip did not produce any torques that are significantly different from controls, but the Lokomat torques applied to the impaired limb are only similar to those applied to the control limb from late stance through early swing. During mid stance the Lokomat produced minimal torques about the hip and yet the impaired limb extended slightly less than the control limb. And as in the unimpaired limb, the impaired limb expressed very low voluntary hip torques, so the Lokomat supplied most of the required hip flexion during late swing.

It should be noted that the results of this study do not indicate individuals following stroke cannot be trained to walk symmetrically in the Lokomat since only one test session was run. Over time, gains in symmetry may be achieved, which has recently been shown in acute stroke subjects following 4 weeks of Lokomat training [21]. The purpose of this study was to simply determine whether stroke subjects guided through a physiological gait pattern would demonstrate symmetric, normal joint torques. Our results indicate that despite symmetric, normal kinematics, the kinetic patterns of stroke subjects are consistent with the stereotypical patterns often exhibited during over-ground walking.

## Conclusion

As would be expected with Lokomat assisted walking, there were very few kinematic differences between the impaired, unimpaired, and control limbs as subjects walked in the device. However, the torques that stroke subjects produce while moving through these patterns are both asymmetrical and quite different from age matched controls. Based on our findings, it appears that hemiparetic stroke subjects cannot break out of stereotypical abnormal motor behaviors in their lower extremities even when the complexity of the task is reduced. Future studies will investigate whether long-term symmetric training with devices such as the Lokomat leads to more symmetric motor patterns and ultimately improved walking ability in hemiparetic stroke subjects.

## Competing interests

The authors declare that they have no competing interests.

## Authors' contributions

NN designed the experiment, carried out the experiments, collected and analyzed the data, and drafted the manuscript. NB prepared subjects and assisted with the experiments. DN prepared subjects and assisted with the experiments. JH designed the experiment, developed the instrumentation and data collection software, and helped draft the manuscript. All authors read, edited, and approved the final manuscript.
